# (Acetonitrile-κ*N*)chloridobis[2-(pyridin-2-yl)phenyl-κ^2^ *C* ^1^,*N*]iridium(III)

**DOI:** 10.1107/S1600536811049373

**Published:** 2011-11-25

**Authors:** Florian Blasberg, Jan W. Bats, Matthias Wagner, Hans-Wolfram Lerner

**Affiliations:** a Institut für Anorganische Chemie der Universität Frankfurt, Max-von-Laue-Strasse 7, D-60438 Frankfurt am Main, Germany; b Institut für Organische Chemie, Universität Frankfurt, Max-von-Laue-Strasse 7, D-60438 Frankfurt am Main, Germany

## Abstract

The Ir^III^ atom of the title compound, [Ir(C_11_H_8_N)_2_Cl(CH_3_CN)], displays a distorted octa­hedral coordination. The pyridyl groups are in *trans* positions [N—Ir—N = 173.07 (10)°], while the phenyl groups are *trans* with respect to the acetonitrile and chloride groups [C—Ir—N = 178.13 (11) and C—Ir—Cl = 176.22 (9)°]. The pyridyl­phenyl groups only show a small deviation from planarity, with the dihedral angle between the planes of the two six-membered rings in each pyridyl­phenyl group being 5.6 (2) and 5.8 (1)°. The crystal packing shows inter­molecular C—H⋯Cl, C—H⋯π(acetonitrile) and C—H⋯π(pyridyl­phen­yl) contacts.

## Related literature

For our work on redox active ligands, see: Jäkle *et al.* (1996 ▶); Guo *et al.* (2001 ▶); Margraf *et al.* (2006 ▶); Kretz *et al.* (2006 ▶); Phan *et al.* (2011 ▶); Scheuermann *et al.* (2008 ▶, 2009 ▶); Blasberg *et al.* (2010 ▶, 2011 ▶). For the synthesis of the starting materials, see: Blasberg *et al.* (2011 ▶); Lowry *et al.* (2004 ▶). For related structures, see: Yang *et al.* (2009 ▶); Shu *et al.* (2011 ▶); McGee & Mann (2007 ▶); Garces *et al.* (1993 ▶).
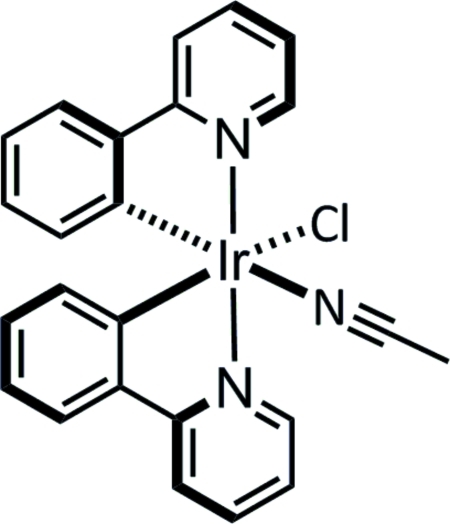


## Experimental

### 

#### Crystal data

[Ir(C_11_H_8_N)_2_Cl(C_2_H_3_N)]
*M* *_r_* = 577.07
Orthorhombic, 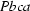

*a* = 16.5255 (8) Å
*b* = 14.6588 (7) Å
*c* = 17.0536 (8) Å
*V* = 4131.1 (3) Å^3^
*Z* = 8
Mo *K*α radiation
μ = 6.61 mm^−1^
*T* = 171 K
0.38 × 0.34 × 0.20 mm

#### Data collection

Siemens SMART 1K CCD diffractometer
Absorption correction: multi-scan (*SADABS*; Sheldrick, 2000 ▶) *T*_min_ = 0.176, *T*_max_ = 0.267
44325 measured reflections
4772 independent reflections
3899 reflections with *I* > 2σ(*I*)
*R*_int_ = 0.043

#### Refinement

*R*[*F* ^2^ > 2σ(*F* ^2^)] = 0.026
*wR*(*F* ^2^) = 0.040
*S* = 1.07
4772 reflections
263 parameters
H-atom parameters constrained
Δρ_max_ = 0.70 e Å^−3^
Δρ_min_ = −0.67 e Å^−3^

### 

Data collection: *SMART* (Siemens, 1995 ▶); cell refinement: *SAINT* (Siemens, 1995 ▶); data reduction: *SAINT*; program(s) used to solve structure: *SHELXS97* (Sheldrick, 2008 ▶); program(s) used to refine structure: *SHELXL97* (Sheldrick, 2008 ▶); molecular graphics: *SHELXTL* (Sheldrick, 2008 ▶); software used to prepare material for publication: *SHELXL97*.

## Supplementary Material

Crystal structure: contains datablock(s) global, I. DOI: 10.1107/S1600536811049373/tk5023sup1.cif

Structure factors: contains datablock(s) I. DOI: 10.1107/S1600536811049373/tk5023Isup2.hkl

Additional supplementary materials:  crystallographic information; 3D view; checkCIF report

## Figures and Tables

**Table 1:** Selected bond lengths (Å)

Ir1—C11	2.004 (3)
Ir1—C22	2.007 (3)
Ir1—N1	2.043 (2)
Ir1—N2	2.047 (2)
Ir1—N3	2.129 (3)
Ir1—Cl1	2.4839 (7)

**Table 2:** Hydrogen-bond geometry (Å, °)

*D*—H⋯*A*	*D*—H	H⋯*A*	*D*⋯*A*	*D*—H⋯*A*
C1—H1*A*⋯Cl1^i^	0.95	2.78	3.600 (3)	145
C14—H14*A*⋯Cl1^ii^	0.95	2.80	3.470 (3)	129
C14—H14*A*⋯C23^iii^	0.95	2.69	3.431 (4)	135
C8—H8*A*⋯C16^iv^	0.95	2.79	3.595 (4)	143
C8—H8*A*⋯C17^iv^	0.95	2.72	3.628 (4)	159
C8—H8*A*⋯C18^iv^	0.95	2.79	3.705 (4)	163

Symmetry codes: (i) 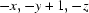; (ii) 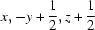; (iii) 
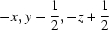
; (iv) 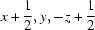.

**Fig. 1.**
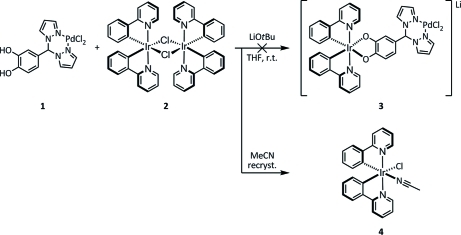
The attempted synthesis of compound 3 and the synthesis of the title compound 4.

**Fig. 2.**
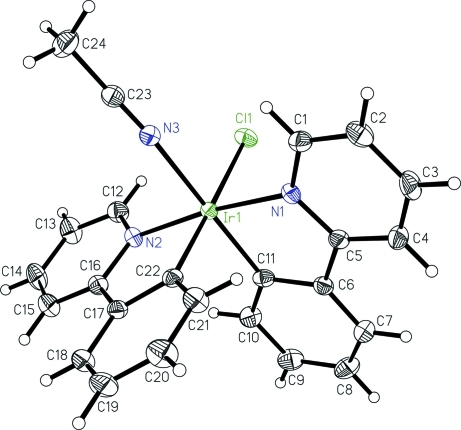
The molecular structure of the title molecule shown with 50% probability displacement ellipsoids. The H atoms are drawn as small spheres of arbitrary radius.

## supplementary crystallographic information

### Comment

One of the highlights of our group's work is the synthesis and characterization of redox active ligands for transition metal catalyzed reactions and applications in material science. Up to now we have applied electro-active ligands with poly(pyrazol-1-yl)borate (Jäkle *et al.*, 1996; Guo *et al.*, 2001), diimine (Margraf *et al.*, 2006; Kretz *et al.*, 2006; Phan *et al.*, 2011) and bis(pyrazol-1-yl)methane (Scheuermann *et al.*, 2008; Scheuermann *et al.*, 2009; Blasberg *et al.*, 2010) donor groups. So far, ferrocenyl and mainly *para*-quinonyl units (quinone is used if the oxidation state is not defined) were used as the redox-active element. But recently our attention turned to *ortho*-quinone derivatives, since they should allow for efficient bridging of two different transition metal centers in redox-switchable catalysis. In this context, synthesis of the hetero-bimetallic complex 3 with an *ortho*-hydroquinone-derived bis(pyrazol-1-yl)methane ligand, a catalytically active palladium(II) and a light-switchable iridium(III) center was attempted (see Fig. 1). This molecule might allow for light-driven redox-reactions, which in turn can switch catalysis on or off. The palladium(II) complex 1 (Blasberg *et al.*, 2010; Blasberg *et al.*, 2011) was deprotonated twice with lithium t-butoxide (LiO*t*Bu) in a tetrahydrofuran solution in a glove box and subsequently tetrakis-[2-(pyridin-2-yl)phenyl]-dichlorido-diiridium(III) 2 (Lowry *et al.*, 2004) was added to the resulting dianion. After stirring overnight and recrystallization of the resulting crude material from acetonitrile, the only obtained product was (acetonitrile-*N*)-chlorido-bis[2-(pyridin-2-yl)phenyl-C,*N*]iridium(III) 4, instead of the expected compound 3.

The molecular structure of the title compound is shown in Fig. 2. The Ir(III) atom displays octahedral coordination (Table 1). The pyridyl groups are in *trans* positions and the phenyl groups in *cis* positions with respect to the central metal atom. A *trans* position of the pyridyl groups also has been observed in dimer 2 (McGee & Mann, 2007; Garces *et al.*, 1993) and in related compounds (Shu *et al.*, 2011). The bond lengths involving the Ir atom are very similar to the values reported for a closely related molecule (Yang *et al.*, 2009). The pyridylphenyl groups only show a small deviation from planarity. The angle between the planes of the two six-membered rings is 5.6 (2) and 5.8 (1)°, respectively, for the two different pyridylphenyl groups. The crystal packing shows two intermolecular C—H···Cl contacts, an intermolecular C—H···π_acetonitrile_ and an intermolecular C—H···π_pyridylphenyl_ contact (Table 2). The C—H···π_acetonitrile_ contact points closer towards atom C23 than towards the midpoint of the C≡ N triple bond. The C—H···π_pyridylphenyl_ contact does not point towards the center of one of the six-membered rings. It rather points towards the midpoint of the C17—C18 bond.

### Experimental

Dichlorido-[1-(bis-1*H*-pyrazol-1-ylmethyl)-benzene-3,4-diol-N,*N*']palladium(II) (50 mg, 0.12 mmol; Blasberg *et al.*, 2011) was reacted with 19 mg (0.23 mmol) of lithium t-butoxide in tetrahydrofuran (4 ml) for 5 min, after which 62 mg (0.12 mmol) of tetrakis-[2-(pyridin-2-yl)phenyl]-dichlorido-diiridium(III) was added. After stirring overnight, the suspension which had formed, was separated by centrifugation and dried by evaporation. Recrystallization of the tan-colored powder from acetonitrile yielded yellow-brown blocks of the title compound.

### Refinement

The H atoms were positioned geometrically and treated as riding with C_planar_—H = 0.95 Å and C_methyl_—H = 0.98 Å, and with *U*_iso_(H)=1.2*U*_eq_(C_planar_) and *U*_iso_(*H*)=1.5*U*_eq_(C_methyl_).

### Crystal data

**Table:**

[Ir(C_11_H_8_N)_2_Cl(C_2_H_3_N)]	*F*(000) = 2224
*M**_r_* = 577.07	*D*_x_ = 1.856 Mg m^−^^3^
Orthorhombic, *P**b**c**a*	Mo *K*α radiation, λ = 0.71073 Å
Hall symbol: -P 2ac 2ab	Cell parameters from 8192 reflections
*a* = 16.5255 (8) Å	θ = 3–26°
*b* = 14.6588 (7) Å	µ = 6.61 mm^−^^1^
*c* = 17.0536 (8) Å	*T* = 171 K
*V* = 4131.1 (3) Å^3^	Block, yellow-brown
*Z* = 8	0.38 × 0.34 × 0.20 mm

### Data collection

**Table:**

Siemens SMART 1K CCD diffractometer	4772 independent reflections
Radiation source: normal-focus sealed tube	3899 reflections with *I* > 2σ(*I*)
graphite	*R*_int_ = 0.043
ω scans	θ_max_ = 28.0°, θ_min_ = 2.2°
Absorption correction: multi-scan (*SADABS*; Sheldrick, 2000)	*h* = −21→21
*T*_min_ = 0.176, *T*_max_ = 0.267	*k* = −18→19
44325 measured reflections	*l* = −21→21

### Refinement

**Table:**

Refinement on *F*^2^	Primary atom site location: structure-invariant direct methods
Least-squares matrix: full	Secondary atom site location: difference Fourier map
*R*[*F*^2^ > 2σ(*F*^2^)] = 0.026	Hydrogen site location: inferred from neighbouring sites
*wR*(*F*^2^) = 0.040	H-atom parameters constrained
*S* = 1.07	*w* = 1/[σ^2^(*F*_o_^2^) + (0.01*P*)^2^ + 6*P*] where *P* = (*F*_o_^2^ + 2*F*_c_^2^)/3
4772 reflections	(Δ/σ)_max_ = 0.002
263 parameters	Δρ_max_ = 0.70 e Å^−^^3^
0 restraints	Δρ_min_ = −0.67 e Å^−^^3^

### Special details

**Table:**

Geometry. All e.s.d.'s (except the e.s.d. in the dihedral angle between two l.s. planes) are estimated using the full covariance matrix. The cell e.s.d.'s are taken into account individually in the estimation of e.s.d.'s in distances, angles and torsion angles; correlations between e.s.d.'s in cell parameters are only used when they are defined by crystal symmetry. An approximate (isotropic) treatment of cell e.s.d.'s is used for estimating e.s.d.'s involving l.s. planes.
Refinement. Refinement of *F*^2^ against ALL reflections. The weighted *R*-factor *wR* and goodness of fit *S* are based on *F*^2^, conventional *R*-factors *R* are based on *F*, with *F* set to zero for negative *F*^2^. The threshold expression of *F*^2^ > σ(*F*^2^) is used only for calculating *R*-factors(gt) *etc*. and is not relevant to the choice of reflections for refinement. *R*-factors based on *F*^2^ are statistically about twice as large as those based on *F*, and *R*- factors based on ALL data will be even larger.

### Fractional atomic coordinates and isotropic or equivalent isotropic displacement parameters (Å^2^)

**Table:**

	*x*	*y*	*z*	*U*_iso_*/*U*_eq_	
Ir1	0.120571 (6)	0.443245 (7)	0.139275 (6)	0.01422 (4)	
Cl1	0.10742 (5)	0.35418 (5)	0.01602 (4)	0.02214 (17)	
N1	0.15302 (14)	0.55553 (17)	0.07570 (14)	0.0168 (5)	
N2	0.09788 (15)	0.33598 (17)	0.21296 (14)	0.0173 (6)	
N3	−0.00690 (15)	0.46420 (16)	0.13721 (15)	0.0180 (5)	
C1	0.10024 (19)	0.6140 (2)	0.04170 (19)	0.0208 (7)	
H1A	0.0442	0.5995	0.0419	0.025*	
C2	0.1253 (2)	0.6939 (2)	0.00676 (19)	0.0271 (7)	
H2A	0.0872	0.7336	−0.0172	0.032*	
C3	0.2071 (2)	0.7156 (2)	0.0070 (2)	0.0269 (8)	
H3A	0.2255	0.7715	−0.0148	0.032*	
C4	0.2612 (2)	0.6548 (2)	0.03935 (19)	0.0227 (7)	
H4A	0.3174	0.6682	0.0389	0.027*	
C5	0.23377 (18)	0.5736 (2)	0.07272 (18)	0.0188 (7)	
C6	0.28432 (19)	0.5020 (2)	0.10731 (18)	0.0183 (7)	
C7	0.3682 (2)	0.5025 (2)	0.10645 (19)	0.0262 (8)	
H7A	0.3960	0.5525	0.0835	0.031*	
C8	0.4118 (2)	0.4314 (2)	0.1385 (2)	0.0314 (8)	
H8A	0.4693	0.4323	0.1378	0.038*	
C9	0.3706 (2)	0.3584 (2)	0.1717 (2)	0.0287 (8)	
H9A	0.4001	0.3090	0.1937	0.034*	
C10	0.2866 (2)	0.3570 (2)	0.17310 (19)	0.0224 (7)	
H10A	0.2595	0.3067	0.1964	0.027*	
C11	0.24108 (18)	0.4280 (2)	0.14108 (18)	0.0184 (6)	
C12	0.07559 (19)	0.2516 (2)	0.1899 (2)	0.0234 (7)	
H12A	0.0702	0.2393	0.1354	0.028*	
C13	0.0605 (2)	0.1831 (2)	0.2425 (2)	0.0286 (8)	
H13A	0.0438	0.1246	0.2249	0.034*	
C14	0.0697 (2)	0.2002 (2)	0.3214 (2)	0.0294 (8)	
H14A	0.0605	0.1531	0.3586	0.035*	
C15	0.0923 (2)	0.2858 (2)	0.34593 (19)	0.0256 (8)	
H15A	0.0991	0.2979	0.4003	0.031*	
C16	0.10527 (18)	0.3545 (2)	0.29141 (18)	0.0183 (7)	
C17	0.12382 (17)	0.4502 (2)	0.30864 (17)	0.0180 (6)	
C18	0.1296 (2)	0.4849 (2)	0.38490 (18)	0.0234 (7)	
H18A	0.1232	0.4452	0.4285	0.028*	
C19	0.1446 (2)	0.5767 (2)	0.3970 (2)	0.0294 (8)	
H19A	0.1488	0.6004	0.4487	0.035*	
C20	0.1534 (2)	0.6340 (2)	0.3325 (2)	0.0298 (8)	
H20A	0.1635	0.6972	0.3403	0.036*	
C21	0.1477 (2)	0.5998 (2)	0.2570 (2)	0.0246 (8)	
H21A	0.1545	0.6400	0.2139	0.030*	
C22	0.13213 (18)	0.5074 (2)	0.24265 (17)	0.0171 (6)	
C23	−0.07549 (19)	0.4705 (2)	0.1405 (2)	0.0216 (7)	
C24	−0.1632 (2)	0.4813 (3)	0.1439 (2)	0.0344 (9)	
H24A	−0.1805	0.5251	0.1038	0.052*	
H24B	−0.1787	0.5039	0.1959	0.052*	
H24C	−0.1892	0.4223	0.1343	0.052*	

### Atomic displacement parameters (Å^2^)

**Table:**

	*U*^11^	*U*^22^	*U*^33^	*U*^12^	*U*^13^	*U*^23^
Ir1	0.01496 (6)	0.01404 (6)	0.01367 (6)	0.00062 (5)	−0.00041 (5)	0.00067 (5)
Cl1	0.0257 (4)	0.0242 (4)	0.0165 (4)	0.0035 (3)	−0.0032 (3)	−0.0025 (3)
N1	0.0193 (12)	0.0163 (12)	0.0148 (12)	0.0023 (11)	0.0007 (10)	0.0020 (12)
N2	0.0202 (14)	0.0168 (13)	0.0149 (13)	0.0008 (10)	0.0016 (10)	0.0009 (11)
N3	0.0201 (14)	0.0152 (13)	0.0186 (13)	−0.0005 (10)	−0.0010 (12)	0.0001 (11)
C1	0.0192 (17)	0.0222 (17)	0.0211 (17)	0.0020 (13)	−0.0020 (13)	−0.0001 (14)
C2	0.0330 (19)	0.0214 (17)	0.0267 (18)	0.0051 (16)	−0.0036 (16)	0.0070 (14)
C3	0.036 (2)	0.0189 (17)	0.0263 (19)	−0.0037 (15)	0.0020 (16)	0.0081 (15)
C4	0.0227 (17)	0.0223 (17)	0.0232 (18)	−0.0061 (14)	0.0014 (14)	0.0016 (14)
C5	0.0189 (16)	0.0225 (18)	0.0149 (15)	−0.0008 (12)	−0.0010 (12)	−0.0017 (13)
C6	0.0201 (16)	0.0193 (16)	0.0156 (16)	−0.0011 (13)	−0.0019 (13)	−0.0003 (13)
C7	0.0222 (18)	0.0309 (19)	0.0255 (17)	−0.0054 (15)	0.0003 (14)	0.0014 (15)
C8	0.0149 (15)	0.042 (2)	0.037 (2)	0.0031 (15)	−0.0031 (16)	0.001 (2)
C9	0.0236 (19)	0.0290 (19)	0.0335 (19)	0.0084 (15)	−0.0064 (16)	0.0032 (15)
C10	0.0241 (17)	0.0168 (17)	0.0263 (18)	0.0010 (13)	−0.0024 (14)	0.0018 (14)
C11	0.0184 (15)	0.0202 (16)	0.0167 (14)	0.0022 (12)	−0.0021 (14)	−0.0059 (15)
C12	0.0259 (18)	0.0228 (18)	0.0216 (19)	−0.0010 (14)	−0.0003 (14)	−0.0030 (15)
C13	0.042 (2)	0.0162 (18)	0.028 (2)	−0.0037 (15)	0.0050 (16)	−0.0002 (15)
C14	0.043 (2)	0.0213 (19)	0.0240 (19)	−0.0031 (16)	0.0048 (16)	0.0089 (15)
C15	0.0321 (18)	0.0248 (18)	0.0200 (19)	−0.0001 (14)	0.0007 (14)	0.0026 (14)
C16	0.0177 (16)	0.0183 (16)	0.0188 (16)	0.0039 (12)	−0.0001 (13)	0.0013 (13)
C17	0.0158 (14)	0.0215 (15)	0.0167 (14)	0.0014 (14)	−0.0013 (12)	−0.0006 (13)
C18	0.0253 (18)	0.0269 (17)	0.0181 (16)	0.0000 (15)	−0.0009 (14)	−0.0004 (13)
C19	0.034 (2)	0.033 (2)	0.0207 (18)	−0.0021 (15)	−0.0022 (15)	−0.0096 (15)
C20	0.040 (2)	0.0200 (18)	0.030 (2)	−0.0014 (15)	−0.0027 (16)	−0.0084 (15)
C21	0.0303 (19)	0.0207 (18)	0.0229 (18)	−0.0002 (14)	−0.0012 (15)	−0.0001 (14)
C22	0.0137 (15)	0.0199 (15)	0.0176 (15)	0.0028 (13)	−0.0011 (12)	−0.0005 (13)
C23	0.0243 (18)	0.0211 (16)	0.0193 (16)	0.0008 (12)	−0.0011 (15)	−0.0014 (15)
C24	0.0205 (18)	0.042 (2)	0.041 (2)	0.0020 (15)	0.0023 (17)	0.003 (2)

### Geometric parameters (Å, °)

**Table:**

Ir1—C11	2.004 (3)	C9—H9A	0.9500
Ir1—C22	2.007 (3)	C10—C11	1.394 (4)
Ir1—N1	2.043 (2)	C10—H10A	0.9500
Ir1—N2	2.047 (2)	C12—C13	1.370 (5)
Ir1—N3	2.129 (3)	C12—H12A	0.9500
Ir1—Cl1	2.4839 (7)	C13—C14	1.376 (5)
N1—C1	1.353 (4)	C13—H13A	0.9500
N1—C5	1.361 (4)	C14—C15	1.374 (5)
N2—C12	1.349 (4)	C14—H14A	0.9500
N2—C16	1.371 (4)	C15—C16	1.388 (4)
N3—C23	1.139 (4)	C15—H15A	0.9500
C1—C2	1.378 (4)	C16—C17	1.466 (4)
C1—H1A	0.9500	C17—C18	1.399 (4)
C2—C3	1.389 (5)	C17—C22	1.410 (4)
C2—H2A	0.9500	C18—C19	1.385 (5)
C3—C4	1.378 (5)	C18—H18A	0.9500
C3—H3A	0.9500	C19—C20	1.392 (5)
C4—C5	1.394 (4)	C19—H19A	0.9500
C4—H4A	0.9500	C20—C21	1.384 (5)
C5—C6	1.465 (4)	C20—H20A	0.9500
C6—C7	1.386 (4)	C21—C22	1.400 (4)
C6—C11	1.422 (4)	C21—H21A	0.9500
C7—C8	1.380 (5)	C23—C24	1.459 (4)
C7—H7A	0.9500	C24—H24A	0.9800
C8—C9	1.389 (5)	C24—H24B	0.9800
C8—H8A	0.9500	C24—H24C	0.9800
C9—C10	1.389 (5)		
			
C11—Ir1—C22	86.81 (12)	C10—C9—H9A	119.7
C11—Ir1—N1	80.64 (11)	C9—C10—C11	121.5 (3)
C22—Ir1—N1	93.65 (11)	C9—C10—H10A	119.3
C11—Ir1—N2	94.97 (11)	C11—C10—H10A	119.3
C22—Ir1—N2	80.69 (11)	C10—C11—C6	117.2 (3)
N1—Ir1—N2	173.07 (10)	C10—C11—Ir1	128.7 (2)
C11—Ir1—N3	178.13 (11)	C6—C11—Ir1	114.1 (2)
C22—Ir1—N3	92.36 (11)	N2—C12—C13	122.0 (3)
N1—Ir1—N3	97.75 (9)	N2—C12—H12A	119.0
N2—Ir1—N3	86.54 (9)	C13—C12—H12A	119.0
C11—Ir1—Cl1	92.36 (9)	C12—C13—C14	119.1 (3)
C22—Ir1—Cl1	176.22 (9)	C12—C13—H13A	120.4
N1—Ir1—Cl1	89.85 (7)	C14—C13—H13A	120.4
N2—Ir1—Cl1	95.72 (7)	C15—C14—C13	119.6 (3)
N3—Ir1—Cl1	88.58 (7)	C15—C14—H14A	120.2
C1—N1—C5	119.5 (3)	C13—C14—H14A	120.2
C1—N1—Ir1	124.6 (2)	C14—C15—C16	120.0 (3)
C5—N1—Ir1	115.7 (2)	C14—C15—H15A	120.0
C12—N2—C16	119.4 (3)	C16—C15—H15A	120.0
C12—N2—Ir1	125.1 (2)	N2—C16—C15	119.8 (3)
C16—N2—Ir1	115.5 (2)	N2—C16—C17	113.8 (3)
C23—N3—Ir1	174.8 (3)	C15—C16—C17	126.4 (3)
N1—C1—C2	122.0 (3)	C18—C17—C22	121.3 (3)
N1—C1—H1A	119.0	C18—C17—C16	123.2 (3)
C2—C1—H1A	119.0	C22—C17—C16	115.4 (3)
C1—C2—C3	119.1 (3)	C19—C18—C17	120.3 (3)
C1—C2—H2A	120.4	C19—C18—H18A	119.9
C3—C2—H2A	120.4	C17—C18—H18A	119.9
C4—C3—C2	118.9 (3)	C18—C19—C20	119.2 (3)
C4—C3—H3A	120.5	C18—C19—H19A	120.4
C2—C3—H3A	120.5	C20—C19—H19A	120.4
C3—C4—C5	120.3 (3)	C21—C20—C19	120.6 (3)
C3—C4—H4A	119.8	C21—C20—H20A	119.7
C5—C4—H4A	119.8	C19—C20—H20A	119.7
N1—C5—C4	120.0 (3)	C20—C21—C22	121.7 (3)
N1—C5—C6	113.8 (3)	C20—C21—H21A	119.1
C4—C5—C6	126.2 (3)	C22—C21—H21A	119.1
C7—C6—C11	120.7 (3)	C21—C22—C17	116.9 (3)
C7—C6—C5	124.2 (3)	C21—C22—Ir1	128.7 (2)
C11—C6—C5	115.1 (3)	C17—C22—Ir1	114.4 (2)
C8—C7—C6	121.0 (3)	N3—C23—C24	178.3 (4)
C8—C7—H7A	119.5	C23—C24—H24A	109.5
C6—C7—H7A	119.5	C23—C24—H24B	109.5
C7—C8—C9	119.1 (3)	H24A—C24—H24B	109.5
C7—C8—H8A	120.4	C23—C24—H24C	109.5
C9—C8—H8A	120.4	H24A—C24—H24C	109.5
C8—C9—C10	120.5 (3)	H24B—C24—H24C	109.5
C8—C9—H9A	119.7		
			
C11—Ir1—N1—C1	176.5 (3)	C22—Ir1—C11—C10	89.2 (3)
C22—Ir1—N1—C1	−97.3 (3)	N1—Ir1—C11—C10	−176.6 (3)
N3—Ir1—N1—C1	−4.4 (3)	N2—Ir1—C11—C10	8.8 (3)
Cl1—Ir1—N1—C1	84.1 (2)	Cl1—Ir1—C11—C10	−87.1 (3)
C11—Ir1—N1—C5	−7.7 (2)	C22—Ir1—C11—C6	−88.3 (2)
C22—Ir1—N1—C5	78.5 (2)	N1—Ir1—C11—C6	5.9 (2)
N3—Ir1—N1—C5	171.4 (2)	N2—Ir1—C11—C6	−168.6 (2)
Cl1—Ir1—N1—C5	−100.1 (2)	Cl1—Ir1—C11—C6	95.4 (2)
C11—Ir1—N2—C12	−98.7 (3)	C16—N2—C12—C13	−0.4 (5)
C22—Ir1—N2—C12	175.4 (3)	Ir1—N2—C12—C13	−179.3 (2)
N3—Ir1—N2—C12	82.4 (2)	N2—C12—C13—C14	−1.2 (5)
Cl1—Ir1—N2—C12	−5.8 (2)	C12—C13—C14—C15	1.2 (5)
C11—Ir1—N2—C16	82.4 (2)	C13—C14—C15—C16	0.4 (5)
C22—Ir1—N2—C16	−3.5 (2)	C12—N2—C16—C15	2.0 (4)
N3—Ir1—N2—C16	−96.5 (2)	Ir1—N2—C16—C15	−179.0 (2)
Cl1—Ir1—N2—C16	175.28 (19)	C12—N2—C16—C17	−175.6 (3)
C5—N1—C1—C2	−2.8 (5)	Ir1—N2—C16—C17	3.3 (3)
Ir1—N1—C1—C2	172.8 (2)	C14—C15—C16—N2	−2.0 (5)
N1—C1—C2—C3	−0.6 (5)	C14—C15—C16—C17	175.3 (3)
C1—C2—C3—C4	2.6 (5)	N2—C16—C17—C18	176.5 (3)
C2—C3—C4—C5	−1.3 (5)	C15—C16—C17—C18	−1.0 (5)
C1—N1—C5—C4	4.2 (4)	N2—C16—C17—C22	−0.8 (4)
Ir1—N1—C5—C4	−171.9 (2)	C15—C16—C17—C22	−178.3 (3)
C1—N1—C5—C6	−176.3 (3)	C22—C17—C18—C19	−0.7 (5)
Ir1—N1—C5—C6	7.7 (3)	C16—C17—C18—C19	−177.8 (3)
C3—C4—C5—N1	−2.1 (5)	C17—C18—C19—C20	0.3 (5)
C3—C4—C5—C6	178.4 (3)	C18—C19—C20—C21	−0.3 (5)
N1—C5—C6—C7	175.6 (3)	C19—C20—C21—C22	0.7 (5)
C4—C5—C6—C7	−4.9 (5)	C20—C21—C22—C17	−1.0 (5)
N1—C5—C6—C11	−2.6 (4)	C20—C21—C22—Ir1	179.5 (3)
C4—C5—C6—C11	176.9 (3)	C18—C17—C22—C21	1.0 (5)
C11—C6—C7—C8	−0.2 (5)	C16—C17—C22—C21	178.3 (3)
C5—C6—C7—C8	−178.3 (3)	C18—C17—C22—Ir1	−179.5 (2)
C6—C7—C8—C9	0.2 (5)	C16—C17—C22—Ir1	−2.1 (3)
C7—C8—C9—C10	−0.3 (5)	C11—Ir1—C22—C21	86.9 (3)
C8—C9—C10—C11	0.4 (5)	N1—Ir1—C22—C21	6.5 (3)
C9—C10—C11—C6	−0.3 (5)	N2—Ir1—C22—C21	−177.5 (3)
C9—C10—C11—Ir1	−177.7 (3)	N3—Ir1—C22—C21	−91.4 (3)
C7—C6—C11—C10	0.2 (5)	C11—Ir1—C22—C17	−92.6 (2)
C5—C6—C11—C10	178.5 (3)	N1—Ir1—C22—C17	−173.0 (2)
C7—C6—C11—Ir1	178.0 (2)	N2—Ir1—C22—C17	3.0 (2)
C5—C6—C11—Ir1	−3.7 (4)	N3—Ir1—C22—C17	89.1 (2)

### Hydrogen-bond geometry (Å, °)

**Table:**

*D*—H···*A*	*D*—H	H···*A*	*D*···*A*	*D*—H···*A*
C1—H1A···Cl1^i^	0.95	2.78	3.600 (3)	145
C14—H14A···Cl1^ii^	0.95	2.80	3.470 (3)	129
C14—H14A···C23^iii^	0.95	2.69	3.431 (4)	135
C8—H8A···C16^iv^	0.95	2.79	3.595 (4)	143
C8—H8A···C17^iv^	0.95	2.72	3.628 (4)	159
C8—H8A···C18^iv^	0.95	2.79	3.705 (4)	163

Symmetry codes: (i) −*x*, −*y*+1, −*z*; (ii) *x*, −*y*+1/2, *z*+1/2; (iii) −*x*, *y*−1/2, −*z*+1/2; (iv) *x*+1/2, *y*, −*z*+1/2.
